# Dual administration of lipopolysaccharide induces behavioural changes in rats relevant to psychotic disorders

**DOI:** 10.1017/neu.2023.40

**Published:** 2023-08-18

**Authors:** Yi-Ran Zheng, Maximilian Tufvesson-Alm, Ada Trepci, Sophie Imbeault, Xue-Qi Li, Lilly Schwieler, Göran Engberg, Sophie Erhardt

**Affiliations:** Department of Physiology and Pharmacology, Karolinska Institutet, Stockholm, Sweden

**Keywords:** psychotic disorders, kynurenic acid, lipopolysaccharide, inflammation, disease models, animal

## Abstract

**Objective:**

We previously reported that dual injections of lipopolysaccharide (LPS) in mice constitute a valuable tool for investigating the contribution of inflammation to psychotic disorders. The present study investigated how immune activation affects the kynurenine pathway and rat behaviour of relevance for psychotic disorders.

**Methods:**

Male Sprague Dawley rats were treated with either dual injections of LPS (0.5 mg/kg + 0.5 mg/kg, i.p.) or dual injections of saline. Twenty-four hours after the second injection, behavioural tests were carried out, including locomotor activity test, fear conditioning test, spontaneous alternation Y-maze test, and novel object recognition test. In a separate batch of animals, in vivo striatal microdialysis was performed, and tryptophan, kynurenine, quinolinic acid, and kynurenic acid (KYNA) in the dialysate were measured using ultra-performance liquid chromatography-tandem mass spectrometry (UPLC-MS/MS).

**Results:**

Dual-LPS treatment decreased spontaneous locomotion, exaggerated d-amphetamine-induced locomotor activity, and impaired recognition memory in male Sprague-Dawley rats. In vivo microdialysis showed that dual-LPS treatment elicited metabolic disturbances in the kynurenine pathway with increased extracellular levels of kynurenine and KYNA in the striatum.

**Conclusion:**

The present study further supports the feasibility of using the dual-LPS model to investigate inflammation-related psychotic disorders and cognitive impairments.

## Significant outcomes

- Dual-lipopolysaccharide (LPS) treatment causes enhanced locomotor response to d-amphetamine, deficits in recognition memory, and anxiety-like behaviour in rats.
- Rats receiving dual-LPS treatment display kynurenine pathway alterations with increased striatal kynurenic acid levels.
- Dual-LPS treatment is a valuable tool for investigating immune-induced-related psychotic disorders.

## Limitations

- Dual-LPS treatment may affect behaviour in rats by altering the level of other neuroactive kynurenine pathway metabolites, such as quinolinic acid (QUIN).
- However, the concentration of QUIN in microdialysis samples was below the limit of quantification and hence not detectable.

## Introduction

Accumulating evidence suggests that immune activation is associated with multiple psychiatric disorders, such as depression (Miller and Raison, 2016; Osimo *et al*., 2020), schizophrenia (Khandaker *et al*., 2015; Orlovska-Waast *et al*., 2019), bipolar disorder (Goldstein *et al*., 2009; Söderlund *et al*., 2011; Rosenblat *et al*., 2015), and anxiety disorders (Vogelzangs *et al*., 2013; Costello *et al*., 2019). Even though the physiological cross-talk between immune activation and psychiatric disorders remains unclear, increasing evidence suggests that the kynurenine pathway of tryptophan degradation may play a crucial pathophysiological role (Savitz, 2020).

The kynurenine pathway constitutes 90–95% of tryptophan degradation in humans and rodents. Notably, pro-inflammatory cytokines activate this metabolism via induction of the rate-limiting enzymes indoldioxygenase 1 and tryptophandioxygenase 2 thereby increasing the production of neuroactive metabolites (Campbell *et al*., 2014; Sellgren *et al*., 2016; Erhardt *et al*., 2017b), such as kynurenic acid (KYNA) and quinolinic acid (QUIN). In this regard, the kynurenine pathway in the brain bridges immune signalling with glutamatergic circuits to induce psychiatric disorders (Erhardt *et al*., 2009; Dantzer *et al*., 2011; Pedraz-Petrozzi *et al*., 2020; Savitz, 2020). Among the kynurenine pathway metabolites, QUIN and KYNA are neuroactive metabolites that have received the most attention. Both metabolites bind to the *N*-methyl-D-aspartate (NMDA) receptor and act as agonists and antagonists, respectively (Stone and Perkins, 1981; Perkins and Stone, 1983; Ganong and Cotman, 1986). Changes in the function of the NMDA receptor are implicated in the pathophysiology of a range of psychiatric disorders (Lakhan *et al*., 2013), especially in mood disorders (Ghasemi *et al*., 2014) and schizophrenia (Balu, 2016). A large body of human studies, all demonstrating elevated levels of central KYNA in patients with schizophrenia (Erhardt *et al*., 2001; Schwarcz *et al*., 2001; Nilsson *et al*., 2007; Sathyasaikumar *et al*., 2011; Linderholm *et al*., 2012; Erhardt *et al*., 2013; Erhardt *et al*., 2017b) and patients with bipolar disorder with a history of psychosis (Olsson *et al*., 2010; Olsson *et al*., 2012b; Lavebratt *et al*., 2014; Sellgren *et al*., 2016; Kegel *et al*., 2017; Sellgren *et al*., 2019; Sellgren *et al*., 2021; Trepci *et al*., 2021), implicate the role of KYNA in psychosis as an executive link between neuroinflammation and NMDA receptor activity.

Administration of the endotoxin lipopolysaccharide (LPS) is an animal model widely used to investigate the role of immune activation in psychiatric diseases (Réus *et al*., 2015). LPS is a pathogen-associated molecular pattern generated from gram-negative bacteria, which induces inflammation by binding to toll-like receptor 4, leading to the release of pro-inflammatory cytokines (Buttini and Boddeke, 1995; Wong *et al*., 1997; Quan *et al*., 1999; Lu *et al*., 2008). In rodents, systemic LPS treatment elicits behavioural abnormalities associated with psychiatric disorders (Yirmiya, 1996; Frenois *et al*., 2007; O’Connor *et al*., 2009; Salazar *et al*., 2012; Oliveros *et al*., 2017; Peyton *et al*., 2019; Imbeault *et al*., 2020; Tufvesson-Alm *et al*., 2020). The behavioural effects of LPS treatment appear to vary depending on the exposure to LPS. A single injection of LPS induces depressive-like behaviours in rodents (Yirmiya, 1996; Frenois *et al*., 2007; O’Connor *et al*., 2009; Salazar *et al*., 2012), whereas dual-LPS treatment elicits aberrant behaviours more relevant to psychotic disorders (Oliveros *et al*., 2017; Peyton *et al*., 2019; Tufvesson-Alm *et al*., 2020). Both single- and dual-LPS treatment induce cognitive impairment in rodents (Pugh *et al*., 1998; Arai *et al*., 2001; Sparkman *et al*., 2005; Oliveros *et al*., 2017; Peyton *et al*., 2019; Imbeault *et al*., 2020; Tufvesson-Alm *et al*., 2020; Mao *et al*., 2021) and activate the kynurenine pathway following induction of indoleamine 2,3-dioxygenase (IDO), but with differential downstream metabolite profiles (O’Connor *et al*., 2009; Larsson *et al*., 2016; Parrott *et al*., 2016a; Parrott *et al*., 2016b; Imbeault *et al*., 2020). Further, LPS exposure, acutely or repeated, promotes neurotoxic kynurenine metabolism in the brain, leading to a rise in the production of 3-hydroxykynurenine and QUIN (Connor *et al*., 2008; O’Connor *et al*., 2009; Parrott *et al*., 2016a; Parrott *et al*., 2016b; Rodrigues *et al*., 2018). On the other hand, dual-LPS injection specifically elicits brain kynurenine metabolism towards KYNA (Oliveros *et al*., 2017; Peyton *et al*., 2019; Tufvesson-Alm *et al*., 2020). Previous studies also show that a reduction in the kynurenine pathway metabolism abrogates the behavioural effects of LPS treatment. Thus, mice deficient in IDO or receiving IDO inhibitors are protected from the behavioural effects of LPS treatment (O’Connor *et al*., 2009; Salazar *et al*., 2012; Heisler and O’Connor, 2015). Moreover, kynurenine monooxygenase knock-out mice are protected from LPS-induced depression-like behaviours (Parrott *et al*., 2016b). Collectively, the divergent behavioural profiles seen in LPS-challenged rodent models may be attributed to a distinct composition of kynurenine pathway metabolites.

Our previous data suggest that dual-LPS treatment is a valuable preclinical model for studying the relation between the dynamic activity of the kynurenine pathway and psychotic disorders in response to immune challenges (Larsson *et al*., 2016; Oliveros *et al*., 2017; Peyton *et al*., 2019; Tufvesson-Alm *et al*., 2020). However, all published studies on the dual-LPS treatment animal model are limited to mice and previous studies suggest species differences between rats and mice with regard to general behaviour and overall regulation of the kynurenine pathway (Fujigaki *et al*., 1998; Allegri *et al*., 2003; Murakami and Saito, 2013; Saré *et al*., 2021). This study aims to investigate in Sprague Dawley rats how immune activation, induced by dual injections of LPS, affects the kynurenine pathway and behaviour of relevance for psychotic disorders.

## Material and method

### Animals, animal welfare, and ethical statement

Experiments were carried out on 200–300 g male Sprague-Dawley rats ordered from Janvier Labs. The rats were housed in groups of 2–4 with free access to water and food. Animal tests were carried out at Komparativ Medicin – Biomedicum, Karolinska Institutet. Environmental conditions were checked daily and maintained under constant temperature (25°C), and 40–60% humidity in a room with a regulated 12 h light/dark cycle (lights on at 07:00). Experiments were approved and performed according to the guidelines of the Ethical Committee of Northern Stockholm, Sweden (ethical number: 2546-2019). All efforts were made to minimise the number of animals used and optimise their well-being.

### Dual-LPS treatment

**Figure 1.**
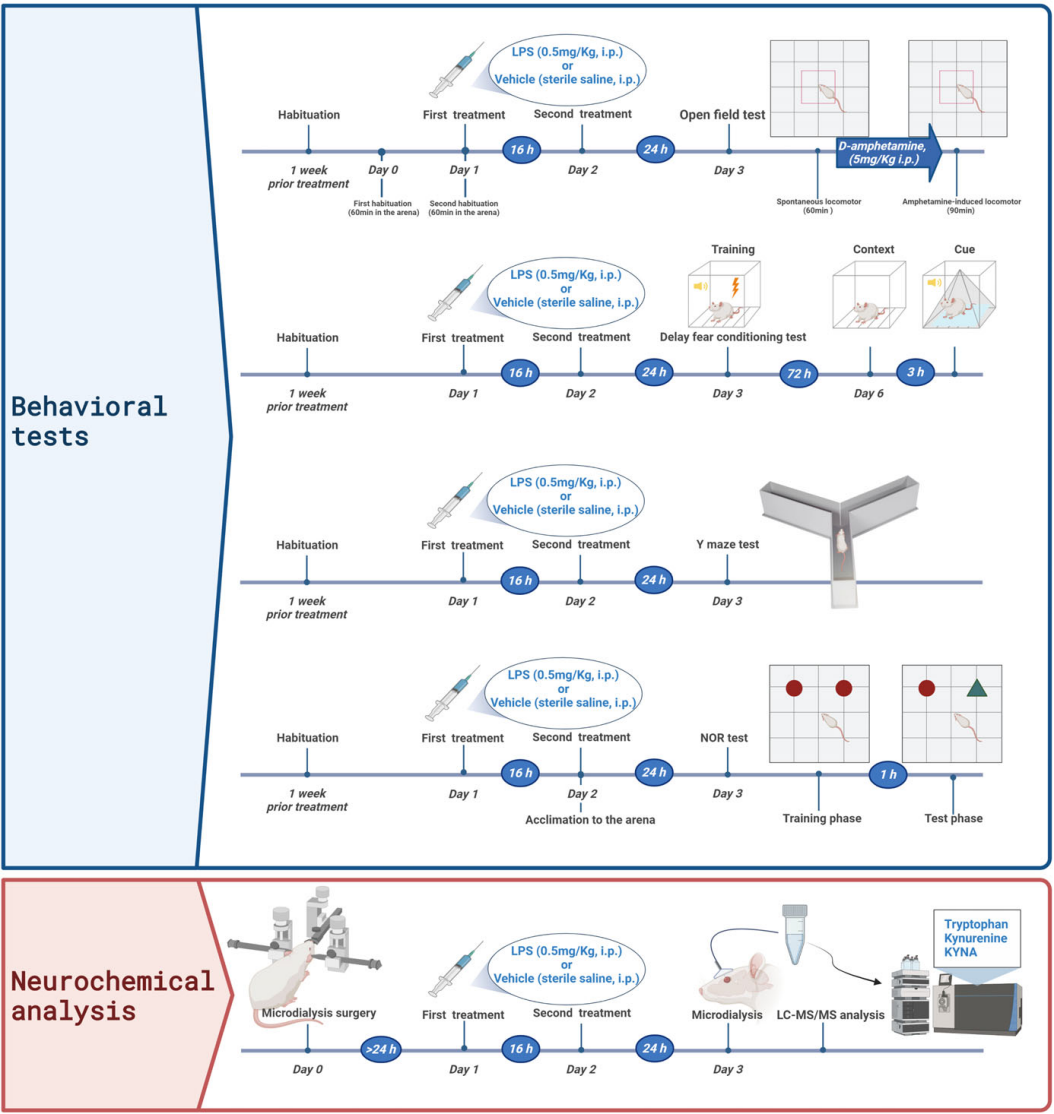
Schematic representation of all the experiments carried out in the present study. Behavioral tests include d-amphetamine-induced hyperlocomotion, fear conditioning, spontaneous alternations in the Y maze, and the novel object recognition (NOR).

Rats were treated with LPS (0.5 mg/kg, i.p.) or equivalent volume vehicle (sterile saline) twice with 16 hours in between. Behavioural assessments as well as in vivo striatal microdialysis were conducted 24 hours after the second injection of LPS or vehicle. In all behaviour tests, the rats were handled for at least 1 min by researchers once a day, starting one week before the tests, to reduce stress. The detailed schedule of each test is shown in Fig. 1. All habituation and testing sessions were conducted between 7 a.m. and 7 p.m.

### Behavioural tests

#### Open-field test

Locomotor activity was assessed in an open-field arena. Each rat was placed in a square Plexiglas box (50 × 50 × 21.6 cm) with a solid, sound-dampened chamber. The chamber was equipped with two rows of photocells that were sensitive to infrared light forming a two-layer grid across the arena (16 cells per row on each side, 3.1 cm apart). A computer recorded each photocell interruption as a single count. Horizontal activity (number of total of beam breaks at the lower level), rearing activity (number of beam breaks at the upper level), and corner time (cumulative time in seconds when two peripheral beams are simultaneously broken) were measured. Rats were habituated following two 60-minute sessions over two consecutive days. Immediately following the second habituation session, animals were treated with LPS or vehicle, as described above. Open field behaviour and basal locomotor activity were assessed 24 hours after the second treatment. After 60 min, rats were immediately injected with either 5 mg/kg d-amphetamine or the same volume of saline, and locomotor activity was recorded during the following 90 minutes. When rats were removed from the chambers, no symptoms of stereotypy were observed. The last 5 minutes of activity before the injection of d-amphetamine was calculated as baseline.

#### Fear conditioning test

We employed contextual plus-cued fear conditioning with a delay fear conditioning paradigm to determine the effect of dual-LPS treatment on associative learning and memory in rats. The test of fear conditioning was conducted using a fear conditioning chamber (Med Associates Inc., St Albans, VT, USA). Training was conducted 24 hours after the second injection of LPS or saline. Rats were given 100 seconds to explore the chamber before introducing a 20-second, 90-decibel tone signal. After the tone was stopped, the foot shock (2 s duration, 0.5 mA intensity) was delivered through the stainless-steel rods on the apparatus’s floor. Foot shock was delivered immediately after the tone, with no gap in time or overlap. After a 100-second inter-trial interval, a second tone-shock pairing was repeated, and the rat was removed 36 seconds after the last shock. Seventy-two hours later, freezing in the same situation was recorded with no tone or shock exposure to evaluate context-dependent memory. Approximately 3 hours after context memory assessment, the rats were again placed in the apparatus. Freezing in response to the cue was recorded in a novel setting (a plastic floor covered the metal grid, and a pyramidal shape was added to the rectangular box). In the cue session, following a 100-second exploration period, the audio cue was provided for 20 seconds. After a 100-second inter-trial interval, the second 20-second cue was provided. All the sessions were automatically recorded by the Med Associate programme (VideoFreeze v.2.5.0.0). Freezing was defined as the absence of movement other than that necessary for breathing. Freezing during each period was scored by experienced observers blinded to treatment.

#### Spontaneous alternation Y-maze test

Twenty-four hours after the second injection of LPS or saline, working memory was assessed using spontaneous alternation in the Y-maze with a classical setting (Swonger and Rech, 1972; Prieur and Jadavji, 2019). Animals were placed in one arm of a Y-maze (each arm had an internal dimension of 50 × 10 × 30 cm) and allowed to freely explore for 8 minutes while being continuously filmed from above. Data were collected using EthoVision XT 16. The parameters used to evaluate the rat performance were calculated using the following formula:

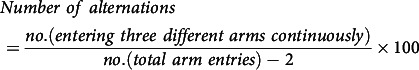




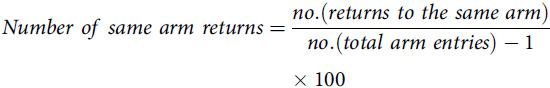




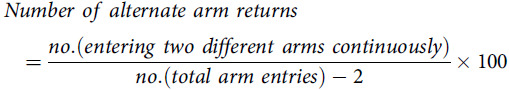




Additionally, the total number of arm entries was computed as an indicator of locomotor activity.

#### Novel object recognition test

The novel object recognition (NOR) test was conducted in an open-field arena (92 × 92 × 40 cm) using a modified previously published protocol (Mathiasen & Dicamillo, 2010) to evaluate recognition memory. The lighting in the test room was controlled at approximately 30 Lx. The procedure consisted of three phases: acclimation, training, and testing. Rats were first acclimated in the arena without objects for 10 min. Twenty-four hours after acclimation, the rats were returned to the arena with two identical objects and given 5 min to freely explore the arena. The test session took place 1 hour after training, during which one of the training objects was replaced with a novel object in the same position. A digital video tracking system (EthoVision XT 16) was used to record the training and test sessions. Exploration was defined as the action with the nose point reaching an object at a distance of no more than 2 centimetres or touching it (Ennaceur & Delacour, 1988). The software automatically measured exploration time.

### Quantitative analysis of tryptophan, kynurenine, QUIN, and KYNA in striatal extracellular fluid

A separate batch of rats was used for neurochemical investigation. Striatal microdialysis was applied to collect extracellular fluid in rats following dual-LPS (*n* = 5) or dual-saline treatment (*n* = 4).

#### In vivo microdialysis

The rats were anaesthetised with isoflurane. The skull was exposed, and a hole was drilled above the striatum (A/P: + 0.48 mm, M/L: ±3.0 mm). Three additional smaller holes were drilled into the skull, and anchor screws were placed before insertion of the guide cannula. A guide cannula (AT4.9.IC; AgnTho’s AB, Sweden) was then lowered into the striatum (D/V: −3.5 mm) and anchored using dental cement (Dentalonâplus, Heraeus, Hanau, Germany). One day after the surgery, rats received either saline or LPS treatment. Twenty-four hours after the second LPS injection, rats were briefly sedated with isoflurane to insert a microdialysis probe (AT4.9.2., shaft length: 9 mm, membrane length: 2 mm, 6 kD cut-off, PES membrane, AgnTho’s AB, Sweden) into the guide cannula. The probe was connected to the microinfusion pump (Univentor 864, Univentor Ltd, Zejtun, Malta) using polyethylene tubing with a constant flow (1 µL/min) of perfusion fluid (perfusion fluid CNS, CMA Microdialysis AB, Sweden). Fractions were collected at 60 min intervals using a cooled autosampler for 9 hours, and all samples were stored at −20°C until further analysis. The metabolite concentrations measured in the microdialysis samples were not corrected for in vitro probe recovery (10–15%). We used a relatively short 3-day period between probe implantation and sampling to mitigate potential impacts on the results caused by scar tissue formation and an associated inflammatory response mediated by cytokine release (Benveniste and Diemer, 1987; Stenken *et al*., 2010).

#### Samples preparation

Microdialysis samples (30 µL) were mixed with 30 µL of internal standard (IS) working solution (10 µM Tryptophan-*d*_*3*_, 1 µM Kynurenine-*d*_*4*_, 1 µM KYNA-*d*_*5*_ and 1 µM QUIN-*d*_*3*_ in 1% formic acid) for 15 s in LC-MS Certified Clear Glass 12 × 32 mm vials (Waters, PN: 186005662CV) before transfer to an autosampler (set to 4°C) that injected 3.0 µL into the UPLC–MS/MS system.

#### Extracellular tryptophan, kynurenine, QUIN, and KYNA analysis by UPLC-MS/MS

Extracellular tryptophan, kynurenine, QUIN, and KYNA values were analysed by UPLC-MS/MS system using a Xevo TQ-XS triple-quadrupole mass spectrometer (Waters, Manchester, UK) equipped with a Z-spray electrospray interface and a Waters Acquity UPLC I-Class FTN system (Waters, MA, USA). In brief, the MS was operated in electrospray-positive multiple reaction monitoring (MRM) mode with a source temperature of 150°C, capillary voltage of +3.0 kV, desolvation temperature of 650°C, desolvation gas flow rate of 1000 L/h, and detector gain 1. The column used was Acquity HSS T3 2.1 × 150 mm, 1.8 μm (Waters, Product Number [PN]: 186,003,540) at a temperature of 50°C. The two mobile phases were composed of A: 0.6% formic acid in water and B: 0.6% formic acid in methanol (UPLC-MS grade). An isolator column (Waters, 2.1 × 50 mm column, PN: 186,004,476) was installed to retain contaminants from the mobile phase. The run time for each sample was 13.0 min, with the flow rate set at 0.3 mL/min. For each individual analyte, the m/z values for MRM were: tryptophan, 206 > 118; kynurenine, 209 > 94; QUIN, 168 > 78; KYNA, 190 > 116. The m/z values for MRM for each IS were tryptophan-*d*_*3*_, 208 > 118; kynurenine-*d*_*4*_, 213 > 94; QUIN-*d*_*3*_, 171 > 81; KYNA-*d*_*5*_, 195 > 121. Full details and method evaluation can be found in our previous study (Schwieler *et al*., 2020).

### Drugs and chemicals

Lipopolysaccharide (*Escherichia coli* serotype O111:B4, Sigma-Aldrich, lot no.: 091M4031V) was prepared in a vehicle solution (sterile saline) daily and stored at 4°C before every injection. D-amphetamine (Sigma-Aldrich) was prepared in a vehicle solution (sterile saline).

### Statistics

Data are presented as mean ± SEM. Statistical significance was set at *P*-value <0.05. Data were analysed using unpaired *t*-test, repeated two-way ANOVA, or mixed-effects model analysis followed by Bonferroni post hoc analysis to determine the differences over time or between treatments. All statistical analyses were performed using GraphPad Prism version 8.3.0 (GraphPad Software, Inc., CA, USA).

## Results

### Dual-LPS-treated rats show reduced spontaneous locomotor activities and increased corner time

**Figure 2.**
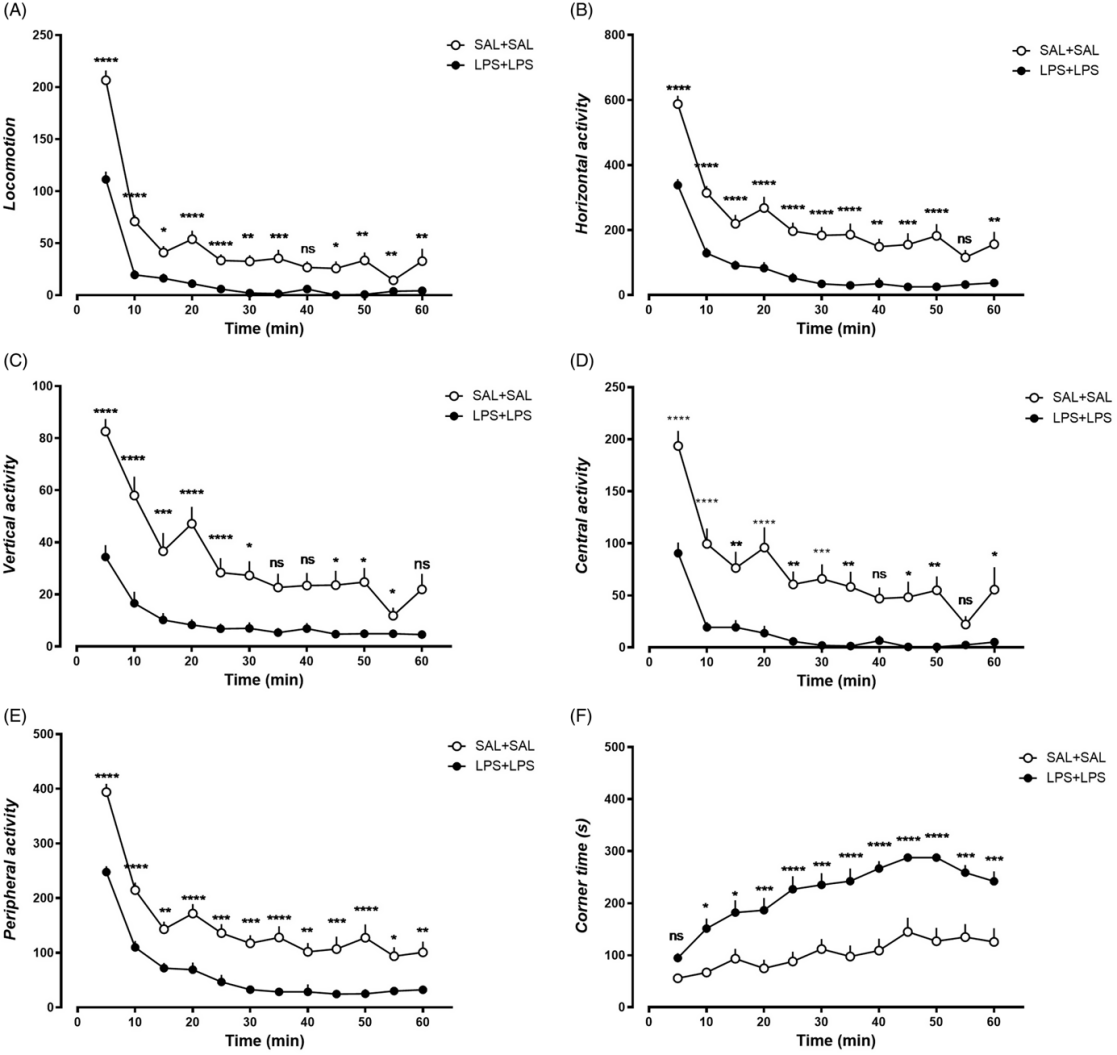
Behavior performance of dual-saline treated rats and dual-LPS treated rats during habituation in the open-field test. A) Locomotion, B) Horizontal activity, C) Vertical activity, D) Center activity, E) Peripheral activity, F) Corner time. N = 20 in each treatment group. * *p* < 0.05, ** *p* < 0.01, *** *p* < 0.001, **** *p* < 0.0001 comparison between groups, Bonferroni post-hoc analysis.

Significant effects of time and treatment were observed in all the measured parameters (Supplementary Table 1). Rats in both treatment groups showed habituation along with session progress (Fig. 2A-E), except for the increasing corner time with session progress (Fig. 2F). Compared to rats treated with dual-saline, rats treated with dual-LPS showed decreased spontaneous locomotor activity with reduced locomotion, horizontal activity, vertical activity, central activity, and peripheral activity (Fig. 2A-E). Moreover, dual-LPS-treated rats showed anxiety-like behaviours reflected as increased time spent in corners compared to saline control (Fig. 2F). A significant effect of interaction between the time and treatment was observed in all measurements of locomotor activities (Supplementary Table 1), with the exception of peripheral activity (Supplementary Table 1).

### Enhanced locomotor response to d-amphetamine following dual-LPS treatment in rats

**Figure 3.**
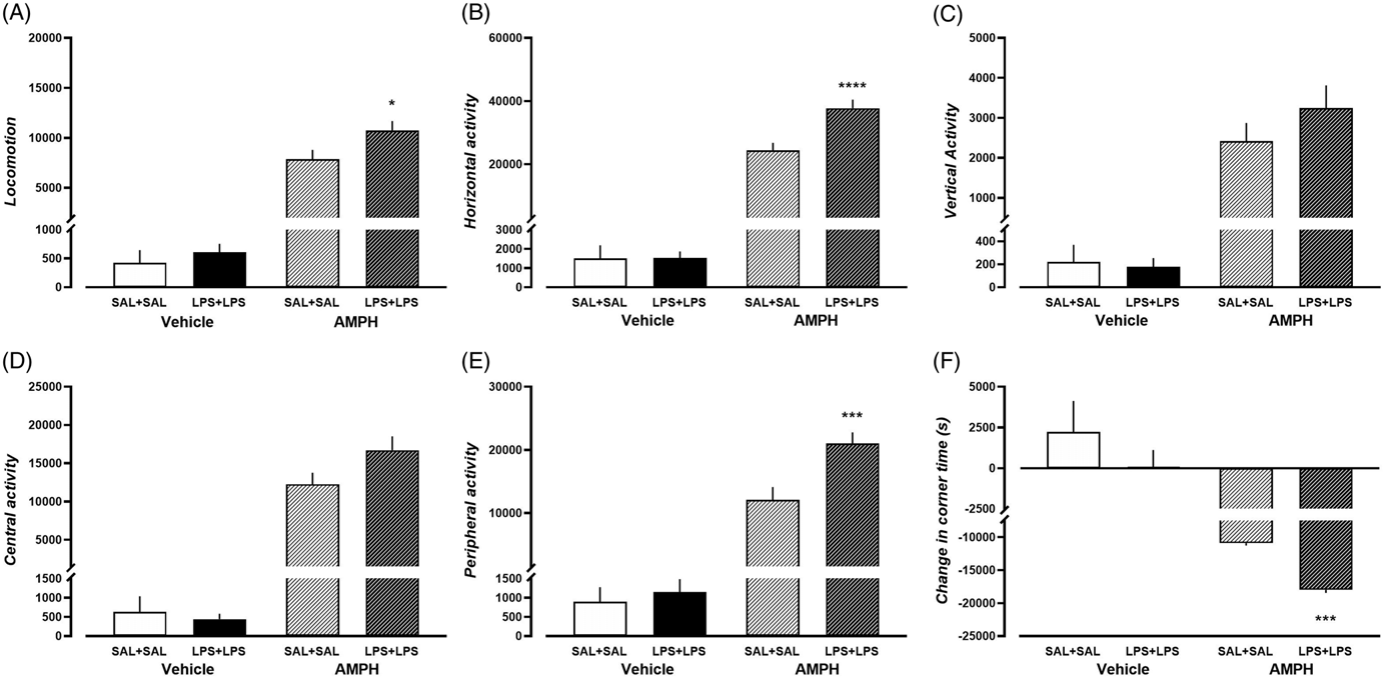
Behavior performance of dual-saline treated rats and dual-LPS treated rats in the d-amphetamine induced locomotor test. Area under the curve (AUC) was measured of A) Locomotion, B) Horizontal activity, C) Vertical activity, D) Center activity, E) Peripheral activity, F) Change in corner time. N = 10 in each treatment group. * *p* < 0.05, *** *p* < 0.001, **** *p* < 0.0001, comparison to the corresponding dual-saline group, Bonferroni post-hoc analysis. AMPH, d-amphetamine.

Following habituation, rats were given either d-amphetamine (5 mg/kg, i.p.) or saline. Locomotor activity was measured for 90 minutes. Given the significantly decreased locomotor activity of dual-LPS-treated rats at baseline (the overall activity of both treatment groups of rats can be found in Supplementary Figure 1), we used the area under the curve to analyse the overall effect of d-amphetamine on the locomotor activity in both groups. D-amphetamine treatment showed the effect of treatment on all the measured parameters (Supplementary Table 2). Dual-LPS-treated rats showed a more robust response to d-amphetamine compared to the dual-saline-treated rats, with enhanced locomotion (Fig. 3A, *p* = 0.0214, *t* = 2.964, df = 36), horizontal activity (Fig. 3B, *p* < 0.0001, *t* = 5.133, df = 36), and peripheral activity (Fig. 3E, *p* < 0.0001, *t* = 4.619, df = 36). Compared to dual-saline-treated rats, rats in the dual-LPS treatment group also showed decreased corner time (Fig. 3F, *p* = 0.0002, *t* = 4.486, df = 36) following d-amphetamine treatment.

### Effects of dual-LPS administration on associative learning and memory

**Figure 4.**
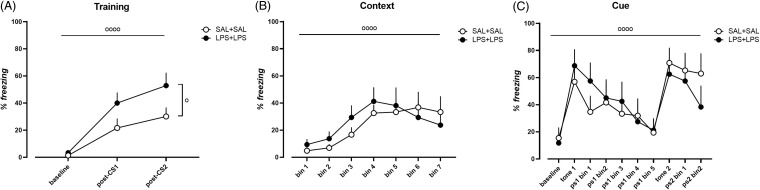
Behavior performance of dual-saline treated rats and dual-LPS treated rats in the delay fear conditioning test. Percent of freezing during A) the training phase, B) context memory test phase, C) the tone-cued memory test phase. N = 9 in the dual-saline treatment group, N= 10 in the dual-LPS treatment group. ^o^ *p* < 0.05, ^oooo^ *p* < 0.0001, repeated measure two-way ANOVA, horizontal line shows effect of training phase, vertical line shows effect of treatment.

We employed contextual plus-cued fear conditioning with a delay fear conditioning paradigm to determine the effect of dual-LPS treatment on associative learning and memory in rats. During the training phase, both dual-saline-treated and dual-LPS-treated rats showed a tone-shock association (Fig. 4A and Supplementary Table 3). In addition, dual-LPS treatment increased freezing in this phase compared with dual-saline-treated animals (Fig. 4A and Supplementary Table 3), indicating anxiety-like behaviour. Seventy-two hours post-training session, both dual-saline-treated and dual-LPS-treated rats exhibited freezing in this context (Fig. 4B and Supplementary Table 3) as well as tone-cued fear conditioning (Fig. 4C and Supplementary Table 3). However, dual-LPS treatment did not change performance in the contextual (Fig. 4B and Supplementary Table 3) or cued conditioning tests (Fig. 4C and Supplementary Table 3).

### Effects of dual-LPS treatment on spatial working memory

**Figure 5.**
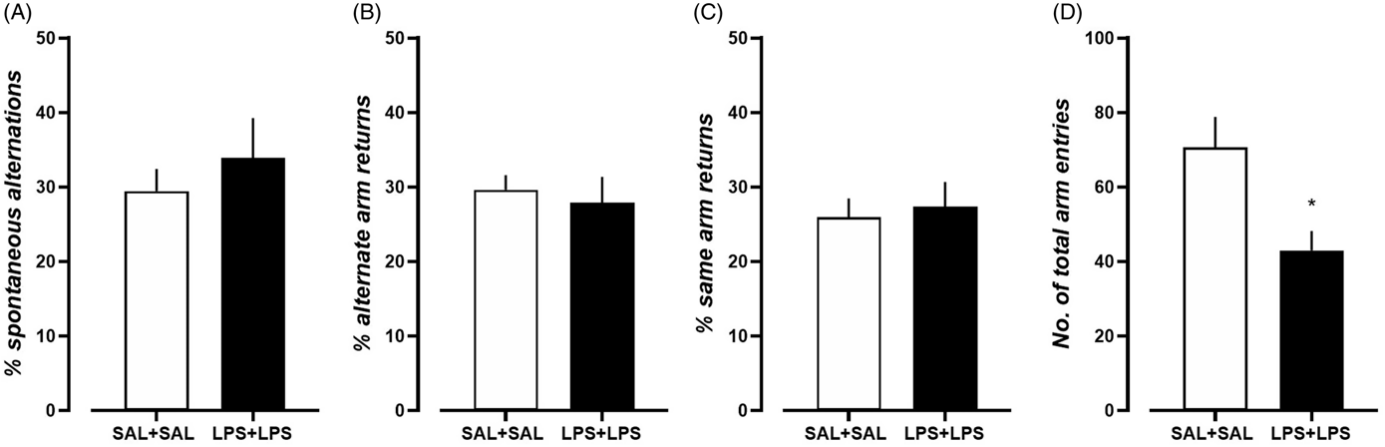
Behavior performance of dual-saline treated rats and dual-LPS treated rats in the spontaneous alternation Y maze test. A) % spontaneous alternations, B) % alternate arm returns, C) % same arm returns, D) number of total arm entries. N = 8 in each treatment group.*p < 0.05, comparison between groups, unpaired *t*-test.

In the Y-maze paradigm, dual-LPS treatment failed to induce any significant changes in spontaneous alteration (Fig. 5A, *p* = 0.4790, *t* = 0.7273, df = 14), alternate arm returns (Fig. 5B, *p* = 0.6681, *t* = 0.4379, df = 14), or same arm returns (Fig. 5C, *p* = 0.7355, *t* = 0.3447, df = 14) when compared with dual-saline treatment. However, reduced locomotion was observed in the dual-LPS-treated rats, as reflected by a reduced number of total arm returns (Fig. 5D, *p* = 0.0134, *t* = 2.827, df = 14).

### Dual-LPS treatment impairs recognition memory in the rat

**Figure 6.**
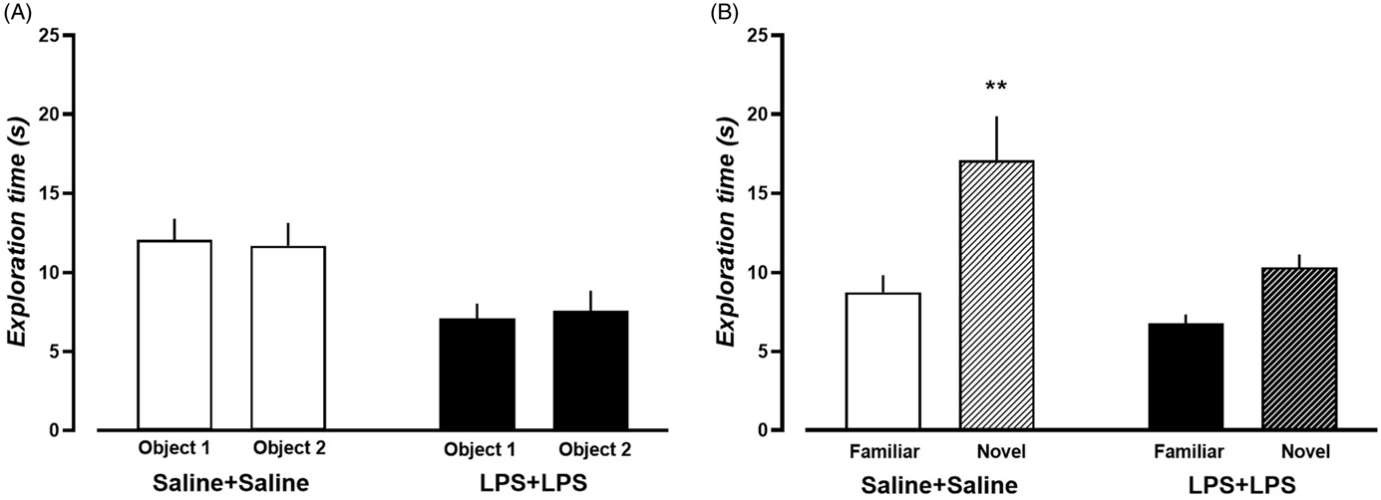
Behavior performance of dual-saline treated rats and dual-LPS treated rats in the novel object recognition test. A) Average exploration time spent on two identical objects during the training phase B) Average exploration time spent on the familiar and novel objects during the test. N = 11 in each treatment group. **p < 0.01, within treatment group comparison between novel and familiar object, Bonferroni post-hoc analysis.

Here we evaluated the recognition memory of dual-LPS-treated rats and dual-saline-treated rats using NOR tests. During the training sessions, for both saline and LPS rats, no significant differences were found in exploration time between the two identical objects (Fig. 6A and Supplementary Table 4). However, compared to saline-treated rats, dual-LPS-treated rats showed a shorter total exploration time (Fig. 6A and Supplementary Table 4). In the test phase, significant effects of object and treatment were observed on the exploration time (Supplementary Table 4). Dual-saline-treated rats spent significantly more time with the novel object than with the familiar one (Fig. 6B, *p* = 0.0023, *t* = 3.785, df = 20). Dual-LPS-treated rats showed no difference in the exploration time between novel and familiar objects (Fig. 6B, *p* = 0.2455, *t* = 1.611, df = 20).

### Increased striatal extracellular kynurenine and KYNA following dual-LPS treatment

**Figure 7.**
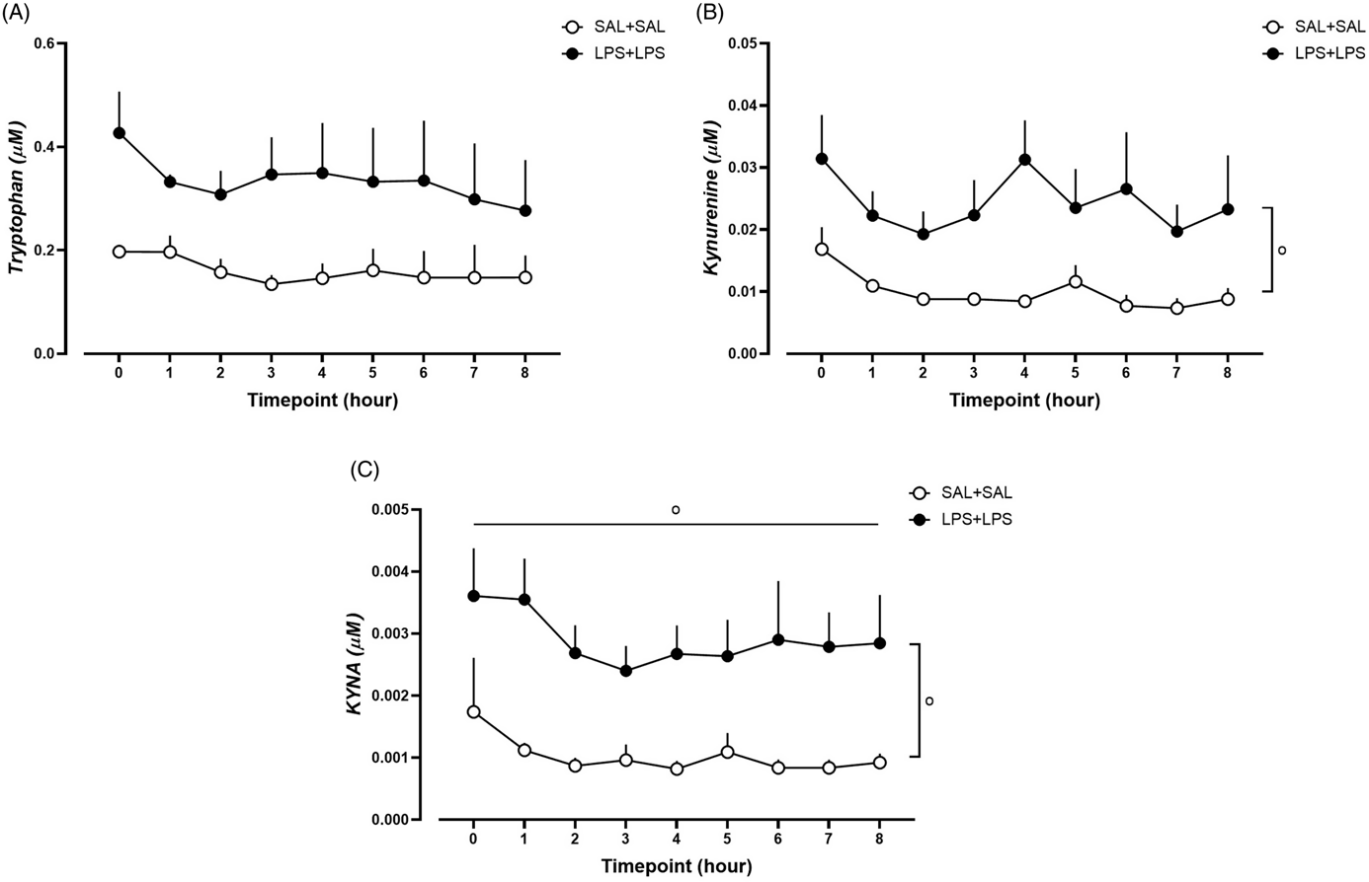
Striatal extracellular fluid A) tryptophan, B) kynurenine, and C) kynurenic acid (KYNA) levels following dual-saline or dual-LPS treatment in rats. N = 4 in the SAL + SAL group, N= 5 in the LPS + LPS group. ^o^ *p* < 0.05, mixed-effects models analysis, horizontal line shows effect of time, vertical line shows of treatment.

Ultra-performance liquid chromatography-tandem mass spectrometry data showed that striatal extracellular levels of tryptophan, kynurenine, and KYNA, measured in the microdialysis samples, were higher than the lowest level of quantification (LOQ: tryptophan, 0.01 μM; kynurenine, 0.00025 μM; KYNA, 0.0005 μM). However, striatal extracellular levels of QUIN were found to be lower than the LOQ (0.005 μM). Thus, due to the limit of assay sensitivity, we could only quantitatively analyse the levels of tryptophan, kynurenine, and KYNA in the microdialysis perfusates. Dual-LPS-treated rats showed increased striatal extracellular kynurenine and KYNA levels compared with saline-treated rats (Fig. 7B-C and Supplementary Table 5). No difference in striatal extracellular tryptophan levels was found (Fig. 7A and Supplementary Table 5).

## Discussion

Previously we reported behavioural effects of dual-LPS treatment in mice by providing solid evidence that such treatment is a promising animal model for studying psychotic disorders with an underlying immune-activated kynurenine pathway, as observed in schizophrenia and bipolar disorder (Larsson *et al*., 2016; Oliveros *et al*., 2017; Peyton *et al*., 2019; Tufvesson-Alm *et al*., 2020). The present study shows that dual-LPS treatment induces aberrant behaviour and cognitive dysfunction of relevance for psychotic disorders in rats. In addition, we show that dual-LPS treatment elicited metabolic alterations in the kynurenine pathway with increased extracellular kynurenine and KYNA levels in the striatum.

Our results with dual-LPS treatment agree with those of previous studies showing that systemic single-LPS treatment reduces spontaneous locomotor activity and induces anxiety-like behaviours in rodents (Bluthé *et al*., 1992; Yirmiya *et al*., 1994; Bison *et al*., 2008; Salazar *et al*., 2012; Sulakhiya *et al*., 2016; Vancassel *et al*., 2022). Notably, anxiety-like behaviour is also observed in mice with elevated brain KYNA, although their spontaneous locomotor activity is decreased or unaffected (Olsson *et al*., 2012a; Erhardt *et al*., 2017a; Tufvesson-Alm *et al*., 2020).

Augmented locomotor response to amphetamine is a well-recognized behavioural abnormality mirroring psychotic symptoms in patients and has repeatedly been observed in validated rodent models of psychotic disorders (Jones *et al*., 2011). In line with our previous data in mice (Tufvesson-Alm *et al*., 2020), dual-LPS-treated rats showed enhanced locomotion, and horizontal, and peripheral activity in response to amphetamine treatment, indicative of a psychotic-like phenotype. However, this contrasts with a recent publication reporting a loss of locomotor response to amphetamine in single LPS-treated mice (Vancassel *et al*., 2022). This discrepancy may confirm the distinct neurobehavioural effects of single versus dual-LPS injections. In this regard, the difference might be related to increased brain KYNA, which is only seen following dual LPS treatment (Larsson *et al*., 2016; Parrott *et al*., 2016b). Indeed, previous studies have shown enhanced amphetamine-induced locomotor activity, likely mediated by dopamine release (Larsson *et al*., 2016), in mice with elevated brain KYNA (Olsson *et al*., 2012a; Erhardt *et al*., 2017a).

Next, we used the fear conditioning test to evaluate associative learning and memory in rats following dual-LPS treatment (Pezze and Feldon, 2004; Curzon *et al*., 2009). Consistent with our previous report in mice (Tufvesson-Alm *et al*., 2020), rats treated with dual injections of LPS displayed enhanced fear acquisition with increased freezing throughout the training session, tentatively indicating anxiety-like behaviours or hypo-locomotor activity.

In contrast to mice (Tufvesson-Alm *et al*., 2020), no differences were observed during the contextual and cued sessions between the dual-saline-treated and dual-LPS-treated rats. The current results suggest intact associative learning and memory in rats following dual-LPS treatment, whereas previous studies describe impaired contextual and cued-dependent fear in LPS-treated mice. Such discrepancies may be species-related. Thus, several previous studies indicate that functional responses to systemic LPS treatment differ between mice and rats (Snyder *et al*., 2009; Lam *et al*., 2017; Saré *et al*., 2021; Genzel, 2021). Moreover, unpublished data from our lab show that dual-LPS treatment affects downstream brain kynurenine metabolism differently in mice and rats, which may account for the behavioural discrepancies observed.

Similar to results obtained in mice (Peyton *et al*., 2019; Tufvesson-Alm *et al*., 2020), dual-LPS-treated rats showed unchanged spontaneous arm alternation performance, indicating intact spatial working memory, in the spontaneous alternation Y-maze test. Our findings contrast with those of previous studies showing spatial working memory deficits following systemic single-LPS injection (Arai *et al*., 2001; Sparkman *et al*., 2006; Murray *et al*., 2012; Zhang *et al*., 2023). Spatial working memory in rodents relies on the interaction of several brain regions, particularly the hippocampus and prefrontal cortex (Swonger and Rech, 1972; Sarnyai *et al*., 2000; Kraeuter *et al*., 2019). The different behavioural patterns induced by single-LPS or dual-LPS treatment may suggest that the exposure time to LPS is critical for affecting brain functioning. Besides, we observed reduced total arm entries in dual-LPS-treated rats during the test, confirming locomotor impairment.

In the assessment phase of the NOR test, dual-saline-treated rats spent more time exploring novel objects, whereas dual-LPS-treated rats spent comparable time exploring familiar and novel objects, indicating impaired recognition memory (Antunes and Biala, 2012). These findings align with those of previous reports, showing that systemic LPS injection impairs recognition memory (Hennigan *et al*., 2007; Frühauf *et al*., 2015; Heisler and O’Connor, 2015; Alzahrani *et al*., 2022). In contrast, a previous report showed that acute LPS treatment does not affect novel object recognition (Czerniawski *et al*., 2015). Of note, in that investigation, LPS was given between the training and the test phase, a design likely to reflect the effects on memory retrieval rather than consolidation (Czerniawski *et al*., 2015). However, in the present study, LPS was administered before training, which would affect both memory consolidation and retrieval. On the other hand, we also need to note that dual-LPS-treated rats already displayed deficits in initial object exploration and spontaneous locomotor activities, which might influence object memory formation.

Dual-LPS rats, like similarly treated mice (Oliveros *et al*., 2017; Peyton *et al*., 2019; Tufvesson-Alm *et al*., 2020), displayed distinct behavioural changes relevant to clinical psychotic disorders and cognitive impairment. We hypothesised that this behavioural phenotype is related to alterations in the brain kynurenine pathway, as suggested in previous studies (O’Connor *et al*., 2009; Heisler and O’Connor, 2015; Parrott *et al*., 2016a; Parrott *et al*., 2016b; Oliveros *et al*., 2017; Peyton *et al*., 2019; Tufvesson-Alm *et al*., 2020). Although one should not ignore the possibility that the implantation probe per se may have affected the inflammatory response by LPS treatment (Stenken *et al*., 2010), we observed increased extracellular kynurenine and KYNA in rat striatum following dual-LPS treatment, which may be attributed to an induction of IDO/TDO and enhanced expression of kynurenine aminotransferase enzymes (O’Connor *et al*., 2009; Larsson *et al*., 2016; Parrott *et al*., 2016a; Parrott *et al*., 2016b). Clinical studies have repeatedly shown that patients with psychotic disorders display centrally elevated KYNA (Erhardt *et al*., 2001; Schwarcz *et al*., 2001; Nilsson *et al*., 2007; Olsson *et al*., 2010; Sathyasaikumar *et al*., 2011; Olsson *et al*., 2012b; Erhardt *et al*., 2013; Lavebratt *et al*., 2014; Sellgren *et al*., 2016; Kegel *et al*., 2017; Sellgren *et al*., 2019; Sellgren *et al*., 2021; Trepci *et al*., 2021), which has been linked to psychotic symptoms (Atlas *et al*., 2007; Olsson *et al*., 2012b; Lavebratt *et al*., 2014) and cognitive dysfunction (Sellgren *et al*., 2016). Studies in rodents demonstrate that experimentally induced elevation of brain KYNA is associated with alterations in glutamatergic and dopaminergic neurotransmission (Erhardt and Engberg, 2002; Schwieler and Erhardt, 2003; Nilsson *et al*., 2006; Linderholm *et al*., 2007; Olsson *et al*., 2009; Pocivavsek *et al*., 2011; Tufvesson-Alm *et al*., 2018), enhanced amphetamine-induced locomotor activity (Olsson *et al*., 2012a; Liu *et al*., 2014; Erhardt *et al*., 2017a), decreased spontaneous locomotor activity (Dennison *et al*., 1992; Chiarugi *et al*., 1995), and impaired cognitive function (Shepard *et al*., 2003; Erhardt *et al*., 2004; Chess and Bucci, 2006; Nilsson *et al*., 2006; Pocivavsek *et al*., 2011). Altogether, it appears likely that the elevation of brain KYNA contributes to the aberrant behaviour seen in rats following dual-LPS treatment.

In developing reliable and predictive animal models for psychotic diseases like schizophrenia and bipolar disorder, corresponding psychosis-related phenotypes are frequently used to understand the neurobiological basis of these conditions (Nestler and Hyman, 2010; Powell and Miyakawa, 2014). However, anxiety symptoms are also highly prevalent in bipolar disorder (Freeman *et al*., 2002) and schizophrenia spectrum psychiatric disorders (Achim *et al*., 2011), and animal models involving anxiety-related phenotypes may offer a more accurate understanding of psychotic disorders (O'Tuathaigh *et al*., 2012). In the present study, a behavioural phenotype not only involving psychosis but also anxiety was validated in dual-LPS-treated rats with enhanced brain kynurenine and KYNA. Thus, the present results confirm that the dual-LPS injection model shows face and construct validity as an animal model of psychotic disorders.

In conclusion, given the broader and more complex repertoire of social performance in rats than in mice, the present results add to our knowledge of the control of behaviours by the immune system. Thus, the present data show that systemic dual-LPS treatment of adult male rats induces behavioural changes and alterations of brain kynurenine pathway relevant to psychotic disorders. Dual-LPS treatment decreased spontaneous locomotion, increased sensitivity to amphetamine, impaired recognition memory, and possibly, anxiety-related behaviours. These behavioural changes are likely caused by elevated brain KYNA, a compound that signals immune activation to neural circuits.

## Supplementary material

The supplementary material for this article can be found at https://doi.org/10.1017/neu.2023.40
